# Congenital Tuberculosis in a Neonate: A Case Report and Literature Review

**DOI:** 10.3389/fped.2019.00255

**Published:** 2019-06-21

**Authors:** Jui-Ju Yeh, Sheng-Chieh Lin, Wen-Chuan Lin

**Affiliations:** ^1^Department of Pediatrics, Shuang Ho Hospital, Taipei Medical University, Taipei, Taiwan; ^2^Department of Family Medicine, Shuang Ho Hospital, Taipei Medical University, Taipei, Taiwan; ^3^Department of Pediatrics, School of Medicine, College of Medicine, Taipei Medical University, Taipei, Taiwan; ^4^Infection Control Office, Shuang Ho Hospital, Taipei Medical University, Taipei, Taiwan

**Keywords:** congenital tuberculosis, neonatal fever, T-helper 2 cells, *Mycobacterium tuberculosis* complex, mortality rate

## Abstract

Congenital tuberculosis (TB) is difficult to detect because the disease presents few or no symptoms in the fetus during pregnancy and nonspecific symptoms in neonates. We reviewed 20 cases of congenital TB reported between 2011 and 2017 and report a case of a mother and her 8 days old neonate with congenital TB. In these 21 cases (including our case), the most common clinical presentations were respiratory distress, fever, and hepatosplenomegaly. The most common chest imaging findings were pneumonia, multiple pulmonary nodules, and miliary pattern. The mortality rate of infants with TB was increased ~2.2-fold if their mothers had no symptoms. The case reported herein concerns an 8 days old neonate with the rare presentation of a 2 days history of fever, followed by abdominal distension without respiratory symptoms. Computed tomography (CT) imaging exhibited a large amount of right pleural effusion. Multiple antimicrobial therapies were administered to the neonate; however, his symptoms persisted. Repeat CT was used to identify a progressed disease with multiple nodules over the lung, spleen, and hepatic hilar region. Standard anti-TB medications were prescribed, and the patient recovered gradually. Both gastric lavage and pleural effusion cultures confirmed the diagnosis of TB. The neonate's mother denied any TB contact history and the diagnosis of any medical disease during pregnancy, but she experienced a fulminant course of miliary TB and was admitted to the intensive care unit 24 days postpartum. She died despite receiving anti-TB treatment. In TB-endemic areas, congenital TB should be taken into consideration when neonates develop fever, respond poorly to antimicrobial treatment, and when their mothers deny any TB contact history.

## Introduction

Tuberculosis (TB), a serious public health problem in many countries, has had the highest rates of incidence and mortality among all communicable diseases worldwide for many years. The World Health Organization estimated that, in 2017, more than 1.6 million deaths were attributable to TB and 10 million people developed the disease. More than 90% of people who developed TB were aged ≥15 years, and the prevalence of TB increased with age. Common symptoms of active pulmonary TB in adults include cough with sputum and blood at times, chest pain, weakness, weight loss, fever, and night sweats (1). However, congenital TB has rarely been reported, with only 358 cases reported in the literature up to 1995 (2) and another 110 cases reported between 1996 and 2009 (3). The mortality rate is high in infants (4). Early diagnosis is critical but challenging because of nonspecific symptoms. Herein, we report the case of a neonate who developed congenital TB and his mother who had an unusual presentation of the disease and review 20 cases reported between 2011 and 2017 (a total of 21 cases including our case).

## Case

### Neonate

**Figure 1:**
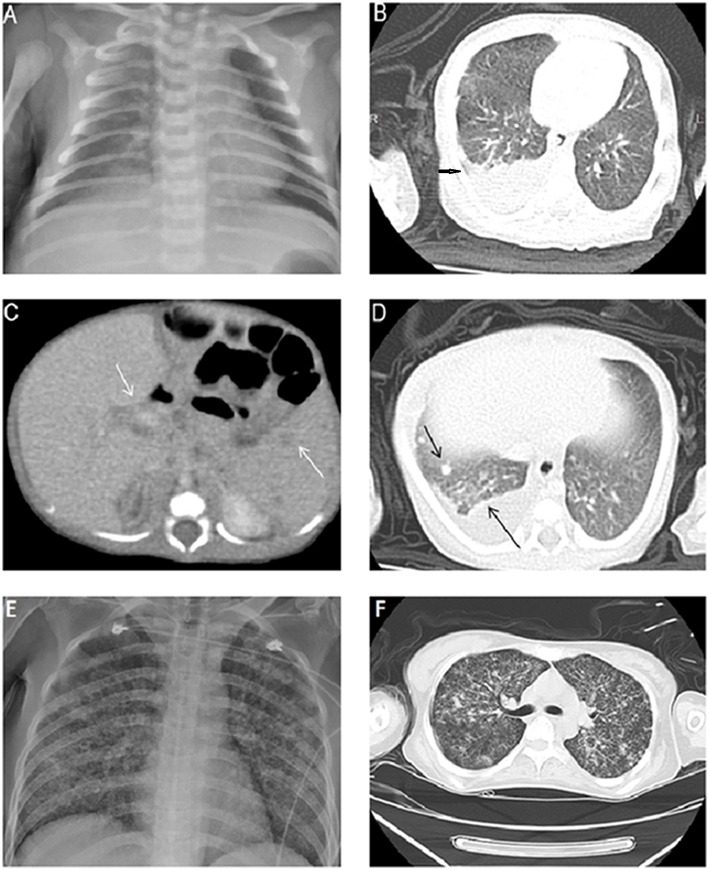
**(A)** Chest X-ray image exhibiting increased bronchoalveolar infiltration over the right lung field. **(B)** Chest CT image depicting a large amount of pleural effusion in the right lung. **(C)** Multiple low-density nodules in the spleen and adenopathy in the hepatic hilar region. **(D)** Multiple new nodules in both lungs and pleurisy. **(E)** Mother's chest X-ray image exhibiting a miliary TB pattern. **(F)** Mother's chest CT image depicting diffuse interlobular and intralobular septal thickening with ground-glass opacities and multiple nodules.

An 8 days old male neonate was born to an Asian mother through vaginal delivery at 37 weeks of gestation, weighed 2,380 g, and had APGAR scores of 9 and 10 at 1 and 5 min, respectively. He was admitted to our hospital with a 2 days history of fever of up to 39°C but did not have respiratory or gastrointestinal symptoms. The infant's family denied any medical history and TB contact. His physical examination at admission documented smooth respiration, clear breathing sound, and no hepatosplenomegaly. The complete blood count indicated a total white blood cell count of 17,500/μL with 69% neutrophils, 20% lymphocytes, 9% monocytes, and 2% eosinophils. The C-reactive protein level was 7.3 mg/dL. The findings of the cerebrospinal fluid (CSF) analysis were normal. Bacterial cultures of the blood, urine, and CSF were negative. Intravenous antibiotics, namely cefotaxime and ampicillin, were administered after admission on the basis of suspicion of neonatal fever. Despite the administration of the antimicrobial combination therapy, the fever persisted and the neonate developed abdominal distension when he was 12 days old. Abdominal radiography exhibited nonspecific dilated bowel loops. Because no improvement in the condition of the patient was observed after changing antibiotics, infection caused by some virus and other atypical pathogen, including *Mycobacterium tuberculosis*, was considered. Tests for herpes simplex virus, Epstein–Barr virus, cytomegalovirus, hepatitis B virus, rubella, *Chlamydia trachomatis*, and *Toxoplasma gondii* were all negative. The repeat C-reactive protein level was elevated to 14.4 mg/dL. Coagulopathy with 323.7 μg/mL of abnormal fibrin degradation product and more than 20 mg/L of D-dimer were also noted. Antibiotics were switched to vancomycin and ceftazidime empirically. Chest radiography displayed only increased right lung field infiltration when the infant was 12 days old (Figure 1A), and chest computed tomography (CT) imaging exhibited a large amount of right pleural effusion with mild inflammatory changes in the right lower lobe when the infant was 15 days old (Figure 1B). Pleural effusion drainage was suggested but refused by his parents at that time. Gastric lavages for acid-fast staining and culture were examined when the infant was 20 days old after his parents agreed to further testing, and one of the three acid-fast stains of gastric lavages yielded few acid-fast *bacilli*. Repeat chest and abdomen CT imaging performed when the infant was 24 days old indicated patchy consolidation in the right upper lung, multiple new nodules in both the lungs, moderate pleural effusion, and multiple low-density nodules in the spleen and hepatic hilar region without hepatomegaly (Figures 1C,D). Subsequently, pigtail catheter insertion for pleural effusion drainage was performed. The findings of pleural fluid analysis indicated a total white blood cell count of 10,800/μL with 6% neutrophils, 57% lymphocytes, and 37% mesothelial cells; a total protein level of 4.6 g/dL, a lactic dehydrogenase level of 250 IU/L, and a glucose level of 164 mg/dL. TB infection was strongly suspected. The neonate was administered isoniazid (15 mg/kg/day), rifampicin (15 mg/kg/day), and pyrazinamide (20 mg/kg/day) when he was 24 days old. After initiating anti-TB treatment, the neonate's symptoms and signs subsided gradually. Finally, both gastric lavage and pleural effusion cultures showed *M. tuberculosis* complex.

### The Neonate's Mother

The neonate's mother was 33 years old, gravida 1, para 1. Her Group B *streptococcus* test was negative. She had been healthy with no previous medical history and TB contact history; however, she developed a mild dry cough 1 week after delivery, experienced persistent general weakness, and was admitted to our medical intensive care unit because of altered mental status 24 days postpartum. Laboratory examinations indicated leukocytosis, thrombocytopenia, coagulopathy, acute hepatic failure, and acute renal failure. The HIV serology test was negative. A chest X-ray exhibited a miliary TB pattern (Figure 1E). A chest CT image displayed diffuse interlobular and intralobular septal thickening with ground-glass opacities (Figure 1F). Because her neonate was highly suspected to have TB infection at that time, acid-fast staining and TB polymerase chain reaction (PCR) of the sputum were performed. Both tests were strongly positive. The mother was administered anti-TB therapy immediately, but she died 3 days after hospitalization. *M. tuberculosis* infection was confirmed through sputum culture.

## Discussion

Congenital TB is rare and can be easily misdiagnosed. Our patient was a neonate with congenital TB with rare presentations. This neonate developed a fever at 6 days old as an initial clinical presentation, and initial chest radiography revealed a large amount of pleural effusion with only mild pulmonary involvement. His family denied that the neonate's mother had TB contact history. TB infection was confirmed through a positive culture of gastric lavages and pleural effusion.

In this study, we reviewed 20 cases of congenital TB reported between 2011 and 2017 on PubMed; of these, 16 cases (80%) were reported in Asian countries (4–23). The male to female ratio was 9:11. The most common chest imaging findings in these 21 cases (including our case) were pneumonia (76%), multiple pulmonary nodules (43%), and miliary pattern (38%). Only three cases presented with liver and spleen lesions, and only two cases presented with pleural effusion. The most common clinical presentations were respiratory distress (71%), fever (67%), hepatosplenomegaly (38%), and cough (33%). Other nonspecific manifestations included poor feeding, lethargy, lymphadenopathy, and seizure. These symptoms and signs were not specific and were easily confused with those observed in other neonatal infections and sepsis. A review reported that the mortality rate was up to 52.6% (1946–1994) and 33.9% (1994–2009) (3). Mothers without symptoms at the time at which initial history was taken constituted 10 of 21 cases (43%), and two of their babies died. The mortality rate of infants with TB was 2.2-fold (20 vs. 9%) higher if their mothers had no symptoms [Tables 1A, 1B; (4–23)]. Our patient was diagnosed with congenital TB. He had clinical presentations of fever followed by abdominal distension without respiratory symptoms, and his imaging findings included pleural effusion and multiple nodules over the lungs, spleen, and hepatic hilar region. *M. tuberculosis* was isolated from gastric lavages and pleural effusion.

**Table 1A:** Most common clinical manifestations of congenital tuberculosis.

**References**	**Symptoms**									**Mother symptoms**	**Infant death**
	**Fever**	**Respiratory distress**	**Hepatic and/or splenic enlargement**	**Lethargy or irritability**	**Poor feeding**	**Cough**	**Abdominal distention**	**Lymphadenopathy**	**Seizures**		
This study	+						+				
Samedi et al. (23)		+								+	
Fang et al. (22)	+		+		+	+				+	
Meyer et al. (21)										+	
Osowicki et al. (20)	+	+				+					
Lee et al. (6)	+	+								+	
Khorsand et al. (14)	+	+		+					+	+	
Chang et al. (4)	+	+								+	
Sagar et al. (13)	+	+	+							+	
Gleeson et al. (11)	+		+								
Hoyos-Orrego et al. (16)		+								+	
Emiralioglu et al. (18)	+	+	+			+	+				
Sen et al. (19)	+	+				+	+				+
Raj et al. (5)	+	+		+	+		+				
Mony et al. (9)		+									
Dewan et al. (10)	+		+	+				+		+	
Espiritu et al. (17)	+	+	+			+				+	+
Zheng et al. (8)	+	+	+			+			+	+	
Singh et al. (12)		+				+					
Nakbanpot et al. (7)		+	+	+	+						+
Grisaru-Soen et al. (15)							+				
Calculated percentage (%)	67	71	38	19	14	33	24	5	10	52	14

**Table 1B:** Most common imaging findings of congenital tuberculosis.

**References**	**Image findings**							
	**Miliary pattern**	**Multiple pulmonary nodules**	**Pneumonia**	**Emphysema**	**Pleural effusion**	**Lesions in the liver/spleen**	**Hepatosplenomegaly**	**Ascites**
This study		+			+	+		
Samedi et al. (23)				+				
Fang et al. (22)		+	+					
Meyer et al. (21)								
Osowicki et al. (20)			+					
Lee et al. (6)	+	+	+		+			
Khorsand et al. (14)			+					+
Chang et al. (4)	+	+	+					
Sagar et al. (13)	+							
Gleeson et al. (11)	+	+	+				+	
Hoyos-Orrego et al. (16)			+					
Emiralioglu et al. (18)	+	+	+				+	
Sen et al. (19)			+					
Raj et al. (5)						+		+
Mony et al. (9)			+					
Dewan et al. (10)			+			+	+	
Espiritu et al. (17)			+				+	+
Zheng et al. (8)	+	+	+				+	
Singh et al. (12)	+	+	+	+				
Nakbanpot et al. (7)	+	+	+					
Grisaru-Soen et al. (15)			+					
Calculated percentage (%)	38	43	76	10	10	14	24	14

Congenital TB can be transmitted during the intrauterine period or during birth. The transmission of congenital TB can be transplacental, where the primary complex is in the liver, or through the aspiration of the infected amniotic fluid or infected material, where the primary complex is in the lung or gut (5). The most common infected organs are the chest and abdominal organs, resulting in fever, cough, respiratory distress, hepatomegaly, jaundice, splenomegaly, and abdominal distension. Other infected organs can include the brain, ear, lymph node, and skin (4). The following diagnostic criteria for differentiating congenital TB from postnatally acquired TB were initially established by Beitzke (5) in 1935: (1) isolation of *M. tuberculosis* from the infant; (2) demonstration of the primary complex in the liver; and (3) in the absence of the primary complex in the liver, (a) evidence of TB within days after birth and (b) absence of contact with TB cases after birth. Beitzke's criteria were later revised by Cantwell (5) in 1994, and the revised criteria are as follows. Proven TB lesions are present in the infant and at least one of the following criteria is met: (1) lesions that occur in the first week of life, (2) caseating granulomas or primary complex in the liver, (3) TB infection of the placenta or maternal genital tract, and (4) a thorough investigation of contacts to exclude postnatal transmission. Inhalation of *M. tuberculosis* by women results in four possible outcomes, namely the immediate clearing of the organism, latent infection, onset of an active disease, or onset of an active disease years later. In published reports, postpartum women have been found to be twice as likely to contract active pulmonary disease or become symptomatic as nonpregnant women (24). Only a few women have been diagnosed with TB during the third trimester of pregnancy. Additionally, some mothers whose infants had active TB remained asymptomatic postnatally (24). Congenital TB is rare but fatal without early diagnosis and treatment. A study of 170 congenital TB cases reported between 1994 and 2009 discovered a high mortality rate; of these cases, a total of 68 patients and patients without treatment died (3). Between 2002 and 2005, four congenital TB cases were reported in Taiwan, and two of them died due to asymptomatic mothers and a delayed diagnosis (4). In our case report, the neonate was saved because of early diagnosis; however, his mother died of miliary TB and multiple organ failure, even after the administration of anti-TB therapy. The proinflammatory response of T-helper 1 (Th1) cells is suppressed and that of T-helper 2 cells increases during pregnancy (24). This status is reversed after delivery. The immune response is similar to that observed in immune reconstitution syndrome. When the level of Th1 cells is elevated, the level of inflammatory cytokines increases. The overwhelming inflammatory response might have aggravated the symptoms of the neonate's mother and contributed to her death.

In Taiwan, the standard treatment of severe extrapulmonary TB in children, including congenital and miliary TB, consists of isoniazid, rifampin, pyrazinamide, and ethambutol in the first 2 months, followed by isoniazid and rifampin for 7–10 months. If a patient is aged <4 years, ethambutol is generally not prescribed because of difficulty monitoring potential optic toxicity.

Congenital TB is treatable if diagnosed and treated early. In areas where it is endemic, it should be taken into consideration, even if a neonate's only symptom is fever. Accurately documenting a maternal history of TB and any clinical symptoms of the disease are critical for early diagnosis. Therefore, particularly in TB-endemic areas, when neonates present with nonspecific symptoms that fail to respond to standard antimicrobial therapy and when postpartum women deny TB contact history but show symptoms of cough with poor activity, the family medical history should be thoroughly examined.

## Ethics Statement

Written informed consent was obtained from the father of the patient for the publication of this case report.

## Author Contributions

JY drafted the initial manuscript and approved the final manuscript as submitted. SL treated and diagnosed the patient, also drafted, reviewed, and revised the manuscript, and approved the final manuscript as submitted. WL is a consultant of infection control, reviewed, and revised the manuscript and approved the final manuscript as submitted. All authors approved the final manuscript as submitted and agree to be accountable for all aspects of the work.

### Conflict of Interest Statement

The authors declare that the research was conducted in the absence of any commercial or financial relationships that could be construed as a potential conflict of interest.

## Abbreviations

TB: tuberculosis
CT: computed tomography
CSF: cerebrospinal fluid
PCR: polymerase chain reaction.

## References

[1] World Health Organization. Global Tuberculosis Report 2018. Available online at: https://www.who.int/tb/publications/global_report/en/ (accessed September 26, 2018).

[2] Hassan G Qureshi W Kadri S Congenital tuberculosis. JK Sci. (2006) 8:193–4.

[3] Peng W Yang J Liu E. Analysis of 170 cases of congenital TB reported in the literature between 1946 and 2009. Pediatr Pulmonol. (2011) 46:1215–24. 10.1002/ppul.21490 21626715

[4] Chang CW Wu PW Yeh CH Wong KS Wang CJ Chang CC. Congenital tuberculosis: case report and review of the literature. Paediatr Int Child Health. (2017) 38:216–9. 10.1080/20469047.2017.1315912 28421876

[5] Raj P Sarin YK Congenital tuberculosis in a neonate: a diagnostic dilemma. J Neonatal Surg. (2014) 3:49. 26023520 PMC4420334

[6] Lee JS Lim CH Kim E Lim H Lee Y Choung JT. Congenital miliary tuberculosis in an 18-day-old boy. Korean J Pediatr. (2016) 59(suppl. 1):S64–7. 10.3345/kjp.2016.59.11.S64 28018449 PMC5177716

[7] Nakbanpot S Rattanawong P. Congenital tuberculosis because of misdiagnosed maternal pulmonary tuberculosis during pregnancy. Jpn J Infect Dis. (2013) 66:327–30. 10.7883/yoken.66.327 23883846

[8] Zheng Y Bai G Zhang H. Congenital tuberculosis detected by T-SPOT.TB assay in a male infant after *in vitro* fertilization and followed up with radiography. Ital J Pediatr. (2014) 40:96. 10.1186/s13052-014-0096-0 25427858 PMC4253620

[9] Mony V Polin J Adler E Munjal I LaTuga M Kojaoghlanian T. Congenital tuberculosis: a missed opportunity. J Pediatric Infect Dis Soc. (2014) 3:e45–7. 10.1093/jpids/piu029 26625463

[10] Dewan P Gomber S Das S. Congenital tuberculosis: a rare manifestation of a common disease. Paediatr Int Child Health. (2014) 34:60–2. 10.1179/2046905513Y.0000000051 24090923

[11] Gleeson L Varghese C Ryan E Kane M McDonald C Gleeson N. Untreated chronic tuberculous salpingitis followed by successfulin vitrofertilization conception and congenital tuberculosis. QJM. (2015) 108:899–901. 10.1093/qjmed/hcv019 25638787

[12] Singh T Natt N Sharma M Singh H. Congenital tuberculosis complicated by interstitial pulmonary emphysema. J Clin Neonatol. (2014) 3:41–3. 10.4103/2249-4847.128731 24741540 PMC3982339

[13] Sagar T Gupta K Rani M Kaur IR. Disseminated tuberculosis in a newborn infant. J Family Med Prim Care. (2016) 5:695–7. 10.4103/2249-4863.197301 28217610 PMC5290787

[14] Khorsand Zak H Mafinezhad S Haghbin A. Congenital tuberculosis: a newborn case report with rare manifestation. Iran Red Crescent Med J. (2016) 18:e23572. 10.5812/ircmj.23572 28184323 PMC5291933

[15] Grisaru-Soen G Savyon M Sadot E Schechner V Sivan Y Schwartz D. Congenital tuberculosis and management of exposure in neonatal and pediatric intensive care units. Int J Tuberc Lung Dis. (2014) 18:1062–5. 10.5588/ijtld.14.0160 25189553

[16] Hoyos-Orrego Á Trujillo-Honeysberg M Diazgranados-Cuenca L. Congenital tuberculosis as a result of disseminated maternal disease: case report. Tuberc Respir Dis. (2015) 78:450–4. 10.4046/trd.2015.78.4.450 26508944 PMC4620350

[17] Espiritu N Aguirre L Jave O Sanchez L Kirwan DE Gilman RH. Congenital transmission of multidrug-resistant tuberculosis. Am J Trop Med Hyg. (2014) 91:92–5. 10.4269/ajtmh.13-0002 24821847 PMC4080578

[18] Emiralioglu N Dogru D Oguz B Yalcin E Ozcelik U Konuskan B. Congenital tuberculosis after *in-vitro* fertilization in a woman previously undiagnosed with tuberculosis salpingitis. Pediatr Neonatol. (2016) 57:539–40. 10.1016/j.pedneo.2015.08.009 27234420

[19] Sen V Selimoglu Sen H Aktar F Uluca Ü Karabel M Fuat Gürkan M. Congenital tuberculosis: presentation of a rare case. Arch Argent Pediatr. (2015) 113:e101–5. 10.5546/aap.2015.e101 25727833

[20] Osowicki J Wang S McKenzie C Marshall C Gard J KeJuin W. Congenital tuberculosis complicated by hemophagocytic lymphohistiocytosis. Pediatr Infect Dis J. (2016) 35:108–10. 10.1097/INF.0000000000000932 26398869

[21] Meyer Sauteur PM Marques-Maggio E Relly C Keller PM Clarenbach CF Berger C. Asymptomatic congenital tuberculosis: a case report. Medicine. (2017) 96:e7562. 10.1097/MD.0000000000007562 28723785 PMC5521925

[22] Fang X Mai R Guo J Lin N A pre-term infant of 32 weeks gestation with congenital tuberculosis treated successfully with antituberculosis chemotherapy. Paediatr Int Child Health. (2017) 14:1–3. 10.1080/20469047.2017.1357879 28805143

[23] Samedi V Field SK Al Awad E Ratcliffe G Yusuf K Congenital tuberculosis in an extremely preterm infant conceived after *in vitro* fertilization: case report. BMC Pregnancy Childbirth. (2017) 17:66 10.1186/s12884-017-1256-1 28219359 PMC5319084

[24] Mathad JS Gupta A. Tuberculosis in pregnant and postpartum women: epidemiology, management, and research gaps. Clin Infect Dis. (2012) 55:1532–49. 10.1093/cid/cis732 22942202 PMC3491857

